# Editorial: Novel strategies to target biofilm formation in ESKAPE pathogens for combating antimicrobial resistance

**DOI:** 10.3389/fmicb.2026.1800825

**Published:** 2026-02-23

**Authors:** Vinothkannan Ravichandran, Aiying Li, Satish Kumar Rajasekharan

**Affiliations:** 1 Amity Institute of Biotechnology, Amity University Maharashtra, Panvel, Maharashtra, India; 2 Deyongshanwei (Guangdong) Biotechnology Co., Ltd., Zhongshan, Guangdong, China; 3 Helmholtz International Lab for Anti-Infectives, State Key Laboratory of Microbial Technology, Shandong University, Qingdao, China; 4 Department of Biotechnology, School of Bioengineering, SRM Institute of Science and Technology, Kattankulathur, Tamil Nadu, India

**Keywords:** antibiofilm strategies, antimicrobial resistance, biofilms, ESKAPEE pathogens, novel antimicrobials

**Figure 1:**
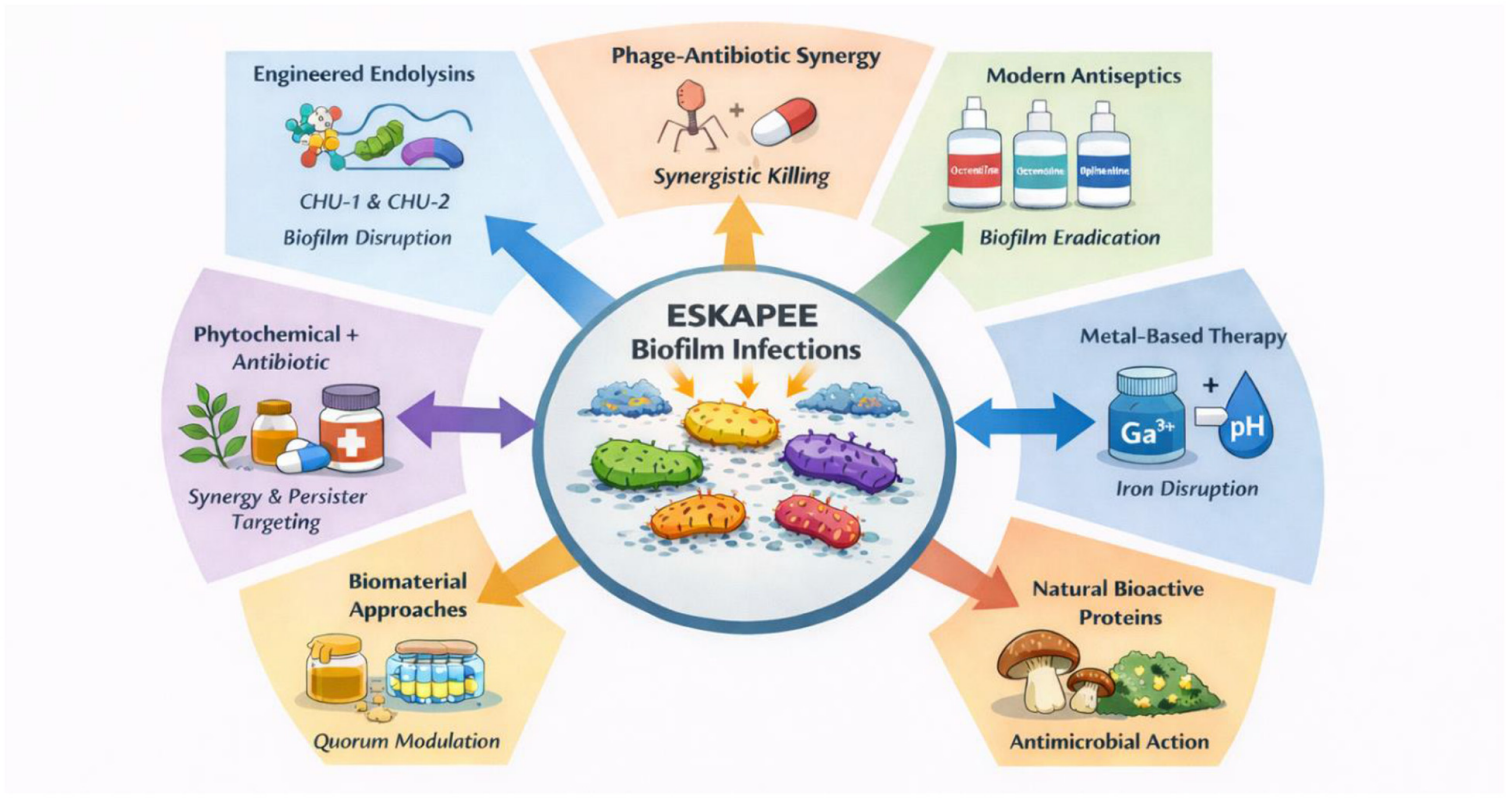
Schematic overview of strategies targeting biofilm-associated antimicrobial resistance in ESKAPEE pathogens.

Antimicrobial resistance (AMR) poses a significant global health risk, primarily due to pathogens that can withstand conventional therapies. Particularly worrisome are the ESKAPE pathogens, which include *Enterococcus faecium, Staphylococcus aureus, Klebsiella pneumoniae, Acinetobacter baumannii, Pseudomonas aeruginosa*, and *Enterobacter* species, are known for their ability to “evade” antibiotics. Infections linked to biofilms formed by these ESKAPE pathogens present a major obstacle in the current medical practice. The formation of structured, multicellular biofilms by these organisms enhances their resistance to antibiotics, disinfectants, and immune responses, thereby playing a crucial role in antimicrobial resistance (AMR), persistent infections, and unfavorable clinical outcomes. Tackling biofilm biology requires strategies extending beyond traditional approaches. This Research Topic brings together eight original articles that collectively showcase the variety, innovation, and translational significance of new antibiofilm strategies targeting ESKAPE pathogens. These studies cover a range of approaches, including engineered biologics, phage-based treatments, synergistic drug combinations, natural products, metal-based therapies, biomaterials, and evaluations of clinically relevant antiseptics (Figure 1).

Chen et al. explored the use of engineered phage-derived endolysins to combat ESKAPE. By combining a *Klebsiella pneumoniae* phage endolysin with ApoE23 and COG133 peptides, they created chimeric proteins that exhibited unique pathogen-specific activity, effectively and swiftly eliminating *A. baumannii, E. faecalis, K. pneumoniae, S. aureus, E. cloacae*, and *P. aeruginosa in vitro*. These optimized constructs demonstrated significant antibiofilm and antipersistent properties, especially against *A. baumannii* and *E. faecalis*, as evidenced by the ultrastructural signs of cell wall damage. Although their effectiveness in a polymicrobial bacteremia model *in vivo* is limited, this study highlights the potential of modular endolysin engineering in developing customized treatments for biofilm-related ESKAPEE infections.

*Staphylococcus aureus*, a notable ESKAPE pathogen, is known for its ability to cause disease, multi-drug resistance (MDR), and potential to cause persistent infections associated with wounds and medical devices. In their study, Muniyasamy and Manjubala evaluated the synergistic effect of the bioactive compounds baicalein and rifampicin. This combined therapy demonstrated greater effectiveness against *S. aureus* biofilms than either treatment alone. These findings indicate that combining phytocompounds with antibiotics can improve antibiofilm and bactericidal effectiveness against *S. aureus*, underscoring their promise in combinatorial therapy.

The interaction between *Staphylococcus aureus* and *Pseudomonas aeruginosa* is known to enhance virulence, increase antimicrobial resistance, and slow wound healing, particularly in foot ulcers. Hilliard et al. investigated the application of Manuka honey, a known antibacterial and wound healing agent, integrated into PCL-gelatin scaffolds. Although soluble manuka honey was effective in inhibiting both planktonic and biofilm growth in co-cultures, the scaffold-embedded version did not directly inhibit or kill bacteria; rather, it promoted agrA expression in *S. aureus*. This study provides a detailed view of how honey-based wound care methods might affect biofilm dynamics, potentially reducing microbial persistence and complications related to wounds in ESKAPE infections.

Liu et al. discovered that gallium nitrate [Ga(NO3)3] can effectively disrupt bacterial iron metabolism by acting like a “Trojan horse.” Because Ga3+ shares the same ionic radius as Fe3+, it is easily absorbed by bacterial iron-acquisition systems but cannot be biochemically reduced, thus hindering crucial iron-dependent functions such as DNA synthesis and electron transport. This study highlights the importance of the environmental pH in determining the effectiveness of this transition metal. Acidic conditions greatly enhanced the vulnerability of *P. aeruginosa* to [Ga(NO3)3]. By identifying pH as a key factor in Ga bioavailability, they introduced a cost-effective and scalable pharmacological approach that supports our aim of developing unconventional treatments to tackle antimicrobial-resistant pathogens.

The widespread occurrence of dental caries continues to be a significant chronic health issue worldwide, impacting billions of people and becoming more complex due to the rise of methicillin-resistant *Staphylococcus aureus* (MRSA) and other MDR oral pathogens. A crucial study conducted by Gangwar et al. investigated an unconventional therapeutic approach by assessing Bioactive Proteins and Peptides (BAPs) derived from both cultivated (*Pleurotus ostreatus*, PoC) and wild (*Pleurotus ostreatus*, PoW) oyster mushrooms. The findings revealed that PoC exhibited stronger antimicrobial properties against *Streptococcus mutans and Lactobacillus acidophilus*, whereas PoW was more effective in preventing biofilm formation by *Enterococcus faecalis*. Both BAP types were effective against MRSA and nystatin-resistant *Candida albicans*, suggesting multiple mechanisms of action. This study advocates a combined pharmacological strategy to treat resistant oral biofilms. Even though some of the organisms examined do not belong to the conventional ESKAPE group, their inclusion is justified by the application of well-established biofilm models and their proven efficacy against ESKAPE-relevant pathogens, such as *Enterococcus faecalis* and MRSA. Mechanistic insights and therapeutic approaches derived from these oral biofilm systems are directly applicable for controlling biofilm-associated infections caused by ESKAPE pathogens.

Vancomycin-resistant *Enterococcus faecalis* (VRE) is responsible for severe hospital-acquired bloodstream infections, with a mortality rate of 20–35% within 30 days, and biofilms make antibiotics ineffective. Wang et al. introduced a groundbreaking approach using the novel lytic phage vB_EfaS-1017, which is the first demonstration of the dose-independent eradication of VRE biofilms. When combined with levofloxacin, this offers an enhanced therapeutic strategy. Analysis of the phage genome confirmed the absence of virulence or resistance genes, thereby ensuring its safety for clinical use. *In vitro* studies showed that vB_EfaS-1017 eliminated 95% of cells within an hour. Importantly, in mouse bacteremia models, phage therapy alone saved 60% of the mice, levofloxacin alone saved 40%, and the combination therapy saved 80%, while reducing phage resistance to 30%, bacterial load, and inflammation. This treatment uniquely restores beneficial gut microbiota, such as *Lactobacillus* and *Alloprevotella*, and provides prophylactic treatment windows, establishing phage-antibiotic synergy as the new gold standard for treating multidrug-resistant enterococcal infections.

*Klebsiella pneumoniae*, a carbapenem-resistant “superbug” and part of the ESKAPE group of pathogens, is responsible for severe hospital-acquired infections in patients with weakened immune systems owing to its biofilm-driven multidrug resistance. Karthika et al. have made progress in phage-antibiotic synergism (PAS) by utilizing a standard phage cocktail consisting of two lytic phages: KPKp (*Ackermannviridae*) and KSKp (*Straboviridae*), in combination with ciprofloxacin (CIP). The small size of CIP allows it to penetrate biofilms, inhibit DNA gyrase activity, and disrupt replication. This cocktail demonstrated extensive lytic activity and prevented escape of resistance through various receptors. At low multiplicity of infection (MOI; 0.001) and sublethal CIP doses ($\frac{1}{8}\times$ MIC), PAS achieved over 90% inhibition of both planktonic and sessile *K. pneumoniae* cells *in vitro*, surpassing the effectiveness of monotherapies. *In vivo*, it significantly prolonged the survival of *Galleria mellonella*, indicating its potential for clinical application.

The ongoing conflict in Ukraine has led to an increase in complex war-related injuries, such as shrapnel wounds and severe burns, which are often further complicated by infections caused by MDR gram-negative bacteria, particularly *Pseudomonas aeruginosa*. This bacterium poses a significant challenge owing to its adaptable metabolism and resistance mechanisms. A key study by Nazarchuk et al. comprehensively evaluated modern antiseptics as essential tools for managing difficult infections. By examining 32 MDR clinical isolates, this study identified a hierarchy of bacteriostatic effectiveness, with decamethoxine and polyhexanide leading the list. Notably, octenidine demonstrated the most potent antibiofilm activity, showing the greatest inhibition of immature biofilms and achieving the highest eradication rate (30.6%) of mature biofilms. Nazarchuk et al. recommend prioritizing octenidine and decamethoxine in surgical protocols to effectively tackle persistent wound infections.

The collection of studies on this Research Topic highlights the necessity of adopting multifaceted and context-sensitive therapeutic approaches to tackle biofilm-related antimicrobial resistance in ESKAPE pathogens. By incorporating engineered endolysins, the synergy between phages and antibiotics, combinations of phytochemicals and antibiotics, metal-based treatments, natural bioactive substances, biomaterial-guided wound strategies, and improved antiseptic application, these studies have moved beyond traditional single-drug therapies toward precision and combination-based solutions. Notably, several contributions have pointed out the discrepancy between *in vitro* success and *in vivo* outcomes, stressing the importance of physiologically relevant models and translational optimization. Collectively, this compilation promotes a shift from indiscriminate bacterial eradication to targeted disruption of biofilm structure, persistence, and polymicrobial interspecies interactions, providing a solid foundation for developing next-generation treatments against stubborn ESKAPE infections.

